# Supplementary material for: A transcriptional patient map of systemic lupus erythematosus reveals disease-related multicellular immune programs conserved between blood and kidney
Source: bioRxiv. 2026 May 1:2026.04.28.721379. Preprint. [Version 1] doi: 10.64898/2026.04.28.721379 (PMC13142532; doi:10.64898/2026.04.28.721379)
Supplement: 2 [file NIHPP2026.04.28.721379v1-supplement-2.pdf]

bioRxiv preprint doi: https://doi.org/10.64898/2026.04.28.721379; this version posted May 1, 2026. The copyright holder for this preprint (which was not certified by peer review) is the author/funder, who has granted bioRxiv a license to display the preprint in perpetuity. It is made available under aCC-BY 4.0 International license.

Supplementary Materials

Supplementary Files

Supplementary File 1.

Sup. Table 1: Explained variance of multicellular programs
Sup. Table 2: Cell-cell communication interactions associated with IFN program. Ligand-receptor (LR) pairs with a Pearson correlation coefficient > 0.3 between their communication activity score (estimated by LIANA+) and IFN program of the multicellular factor model.
Sup. Table 3: Statistics for the archetype association to the IFN program
Sup. Table 4: Cytokine footprint activity differences between SLE and healthy archetypes
Sup. Table 5: Cox proportional hazards models using longitudinal cohort.
Sup. Table 6: Performance metrics from MISTy models to study spatial co-localization of cell-types between healthy and SLE slides

bioRxiv preprint doi: https://doi.org/10.64898/2026.04.28.721379; this version posted May 1, 2026. The copyright holder for this preprint (which was not certified by peer review) is the author/funder, who has granted bioRxiv a license to display the preprint in perpetuity. It is made available under aCC-BY 4.0 International license.

Supplementary Figures

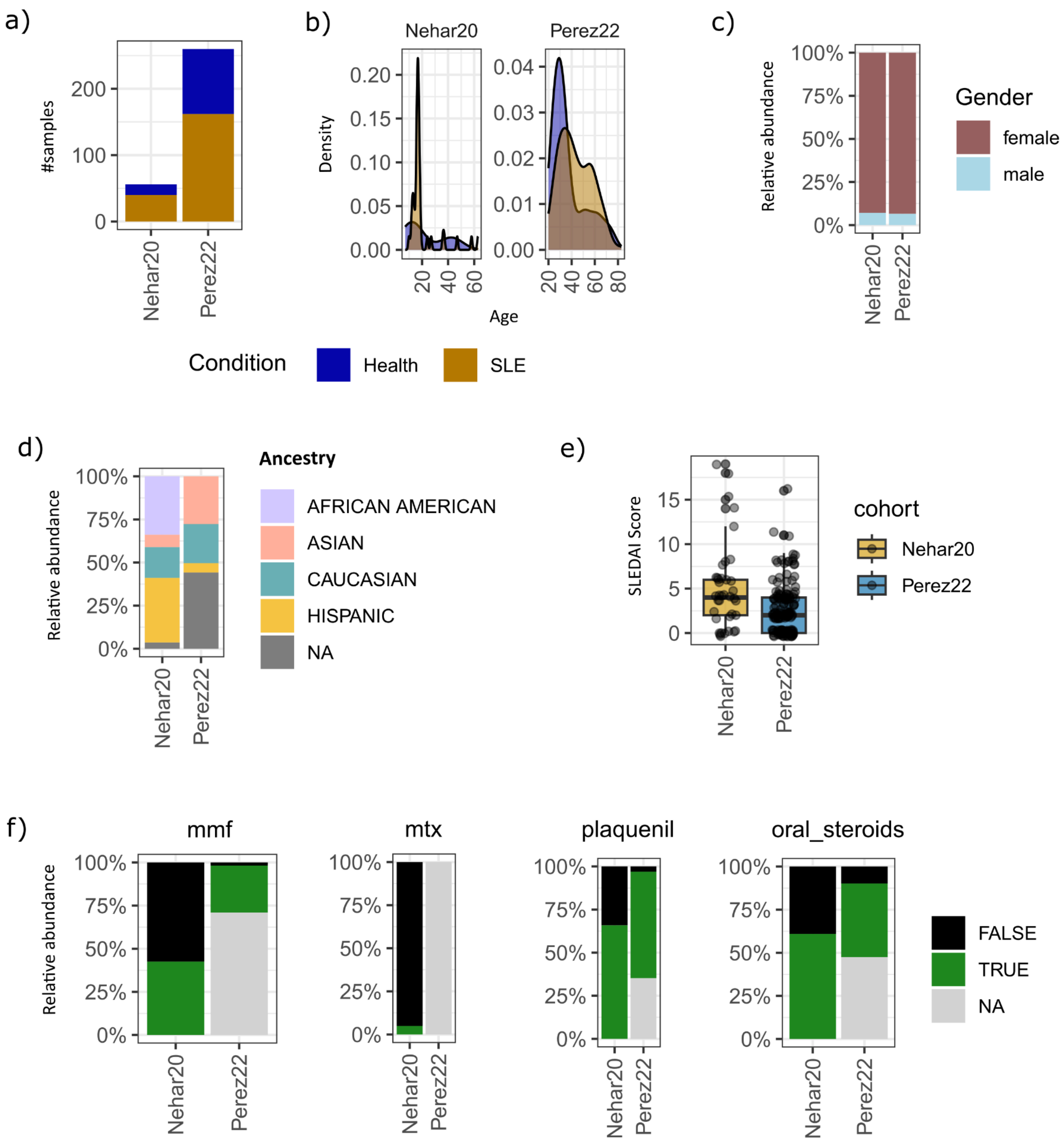

bioRxiv preprint doi: https://doi.org/10.64898/2026.04.28.721379; this version posted May 1, 2026. The copyright holder for this preprint (which was not certified by peer review) is the author/funder, who has granted bioRxiv a license to display the preprint in perpetuity. It is made available under aCC-BY 4.0 International license.

Supplementary Figure S1 - Patient-level clinical and demographic characteristics

(A) Number of samples per cohort (Nehar-Belaid 2020, Pérez 2022) stratified by condition (Healthy, SLE). (B) Age distribution of participants by cohort and condition. (C) Sex distribution across cohorts. (D) Self-reported ancestry composition for individuals with available data. (E) Distribution of disease activity scores (SLEDAI) in SLE patients by cohort. (F) Proportion of SLE patients receiving immunomodulatory treatments, including mycophenolate mofetil (MMF), methotrexate (MTX), hydroxychloroquine (Plaquenil), and oral corticosteroids. Grey indicates missing data.

bioRxiv preprint doi: https://doi.org/10.64898/2026.04.28.721379; this version posted May 1, 2026. The copyright holder for this preprint (which was not certified by peer review) is the author/funder, who has granted bioRxiv a license to display the preprint in perpetuity. It is made available under aCC-BY 4.0 International license.

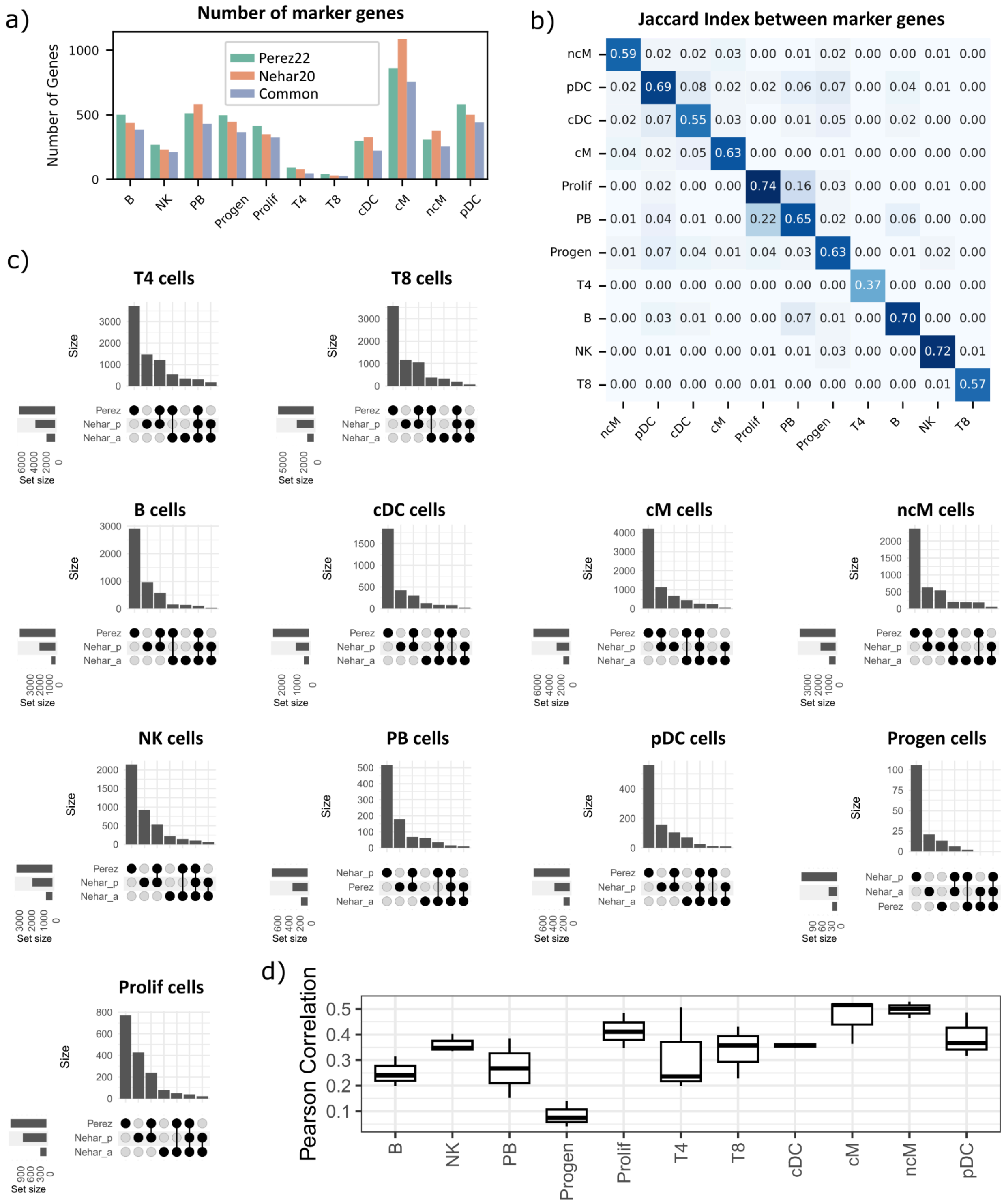

bioRxiv preprint doi: https://doi.org/10.64898/2026.04.28.721379; this version posted May 1, 2026. The copyright holder for this preprint (which was not certified by peer review) is the author/funder, who has granted bioRxiv a license to display the preprint in perpetuity. It is made available under aCC-BY 4.0 International license.

Supplementary Figure S2 - Cross-cohort consistency of cell-type marker and differential expression signatures

(A) Number of marker genes identified per cell type (logFC > 3; P < 0.001). Bars represent markers specific to each cohort (green and orange) and those shared between cohorts (blue). (B) Pairwise Jaccard index heatmap showing the similarity of marker gene sets across all immune cell types between cohorts. (C) UpSet plots displaying intersections of differentially expressed genes (adjusted P < 0.05) identified in SLE versus Healthy comparisons across cohorts. The Nehar-Belaid dataset was divided into pediatric and adult subgroups for independent analysis. (D) Distribution of Pearson correlation coefficients (r) comparing t-statistics from differential expression analyses across cohorts. Each boxplot summarizes three pairwise comparisons (Pérez vs Nehar-pediatric, Pérez vs Nehar-adult, and Nehar-pediatric vs Nehar-adult).

bioRxiv preprint doi: https://doi.org/10.64898/2026.04.28.721379; this version posted May 1, 2026. The copyright holder for this preprint (which was not certified by peer review) is the author/funder, who has granted bioRxiv a license to display the preprint in perpetuity. It is made available under aCC-BY 4.0 International license.

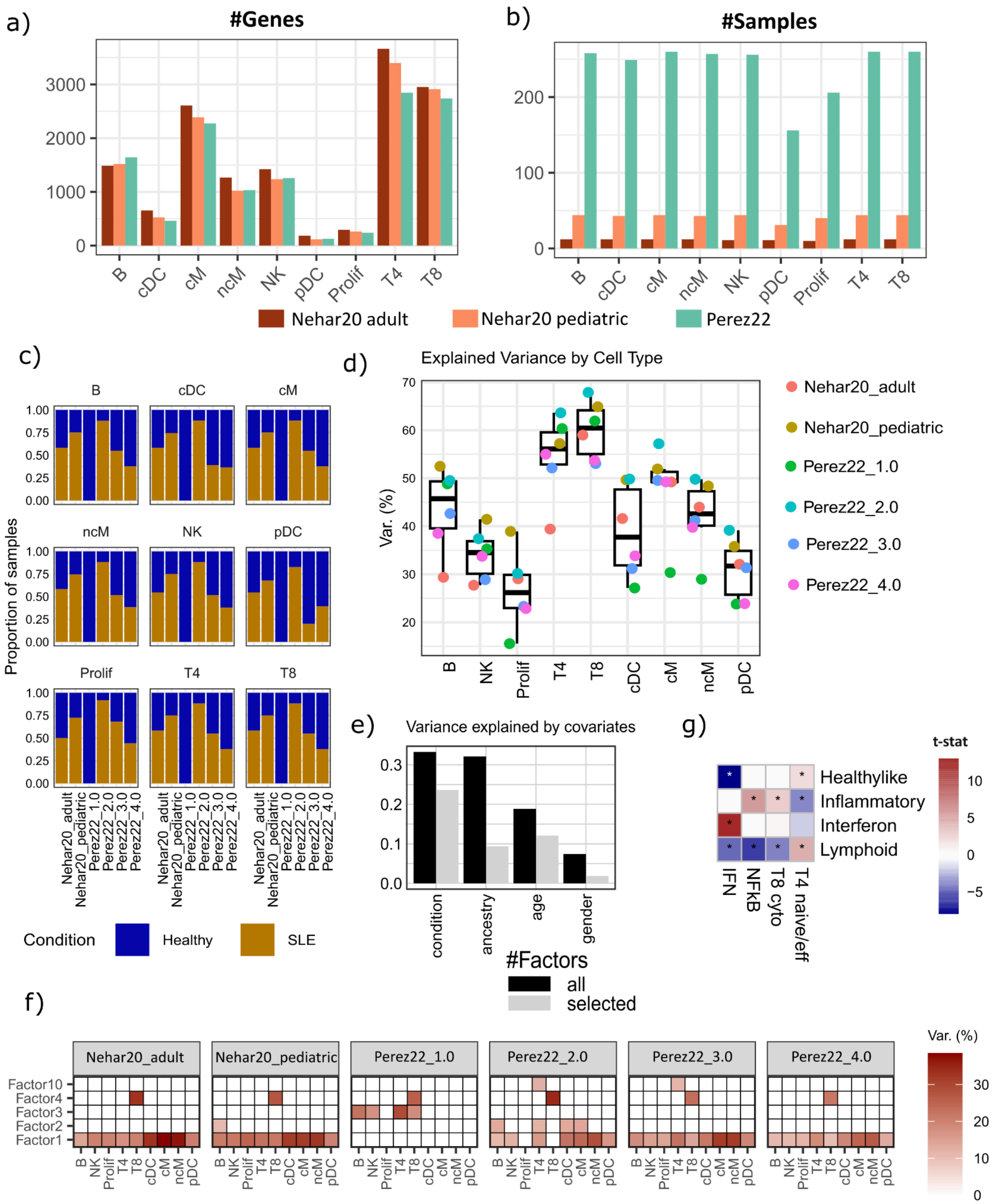

bioRxiv preprint doi: https://doi.org/10.64898/2026.04.28.721379; this version posted May 1, 2026. The copyright holder for this preprint (which was not certified by peer review) is the author/funder, who has granted bioRxiv a license to display the preprint in perpetuity. It is made available under aCC-BY 4.0 International license.

Supplementary Figure S3 - Overview of input data and explained variance of the multicellular factor model

(A) Number of genes per cell type and cohort used as input for the multicellular factor model. (B) Number of samples per cell type and cohort used as input for the multicellular factor model. (C) Proportion of healthy and SLE samples across all cell types and cohorts used as input for the multicellular factor model. (D) Mean explained variance (R^2) per cell type across cohorts after model training. (E) Variance explained by clinical and demographic covariates (condition, ancestry, age, gender, cohort) when considering all latent factors (n = 19) or the selected subset (n = 4), estimated via multivariate linear regression. Factor 3 was excluded, as it predominantly captured variation associated with healthy samples. (F) Explained variance (R^2) across cohorts, cell types, and factors. Only factors explaining ≥10% of the variance in at least one cohort or cell type are shown; others were excluded. For visualization, R^2 values below 10% were set to 0. (G) Mapping multicellular programs onto an external cohort (PRECISESADS, n = 226 SLE patients) and enrichment of these programs in molecular groups defined in the original article (Interferon, Inflammatory, Lymphoid and Healthy-like). Tiles display t-statistics from linear models, with color indicating directionality and asterisks denoting significance (adjusted P val < 0.05)

bioRxiv preprint doi: https://doi.org/10.64898/2026.04.28.721379; this version posted May 1, 2026. The copyright holder for this preprint (which was not certified by peer review) is the author/funder, who has granted bioRxiv a license to display the preprint in perpetuity. It is made available under aCC-BY 4.0 International license.

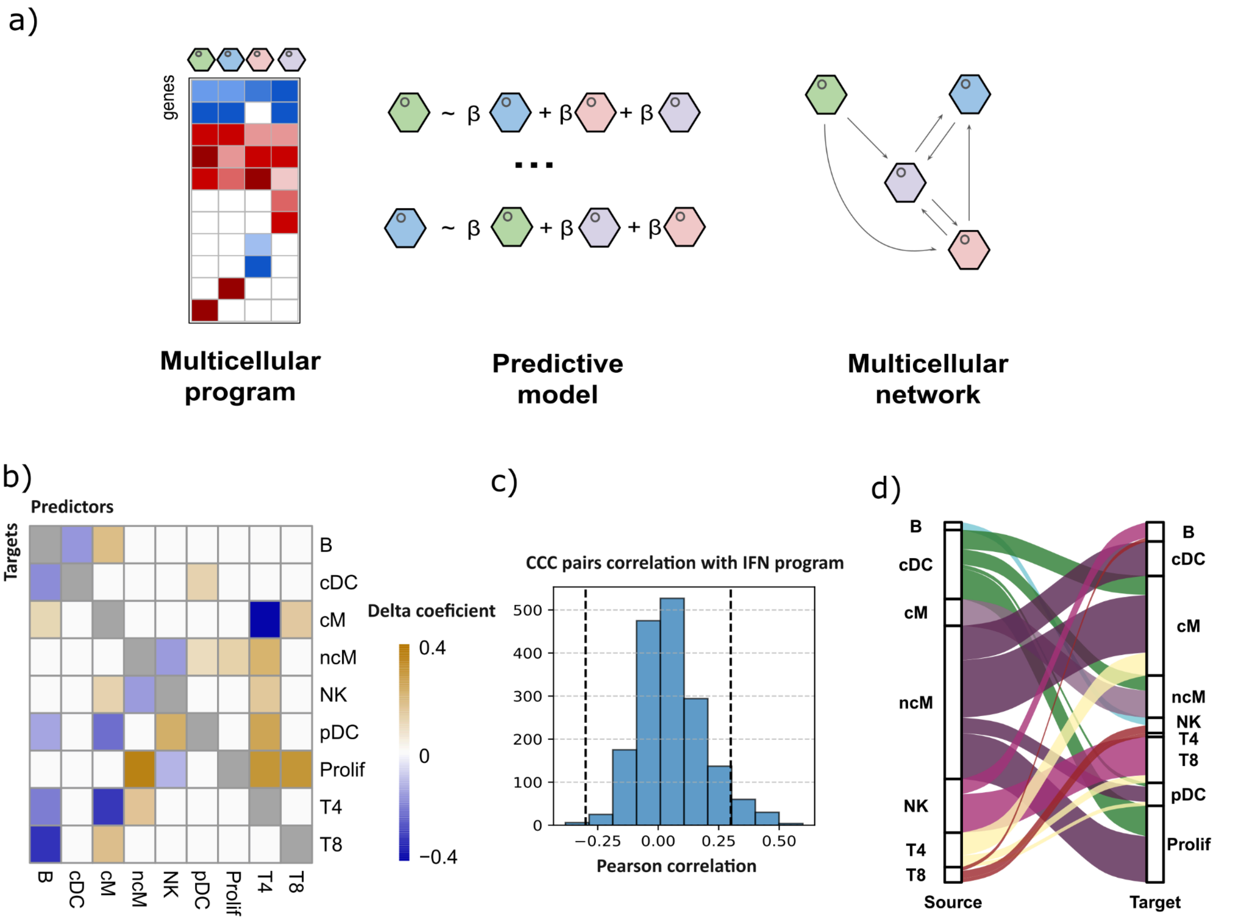

Supplementary Figure S4 - Multicellular coordination and cell-cell communication driven by IFN program.

(A) Schematic overview of the framework used to infer multicellular coordination from pseudobulk transcriptomic profiles. Gene-level variation across immune cell types is decomposed into latent factors that capture shared multicellular programs. Intercellular coordination is quantified by predictive modeling of factor loadings across cell types, followed by inference of ligand–receptor (LR) communication links constrained by the resulting co-expression network. (B) Difference in cell-type dependencies within the IFN program between SLE and healthy samples. Each cell shows the difference in ridge regression coefficients (SLE − healthy) for a given predictor (column) - target (row) pair. Coefficients below a magnitude threshold of 0.1 in either condition were set to zero, and pairs where both conditions shared the same sign were excluded. Orange indicates

bioRxiv preprint doi: https://doi.org/10.64898/2026.04.28.721379; this version posted May 1, 2026. The copyright holder for this preprint (which was not certified by peer review) is the author/funder, who has granted bioRxiv a license to display the preprint in perpetuity. It is made available under aCC-BY 4.0 International license.

dependencies stronger in SLE; blue indicates dependencies stronger in healthy networks. Diagonal elements (self-predictions) are shown in gray. (C) Distribution of pairwise Pearson correlation coefficients (*r*) between Factor 1 loadings and ligand–receptor (LR) interaction activities inferred using LIANA (see Methods). The dashed vertical line denotes $|r| = 0.3$. CCC interactions were constrained to those identified within the merged multicellular network (Healthy + SLE). (D) Summary of significant ligand–receptor (LR)–mediated cell–cell communication (CCC) events along the *Factor 1* axis. Significant interactions were identified based on Pearson correlation ($r > 0.3$, $P < 0.05$) between LR interaction scores and *Factor 1* loadings across individuals. Each interaction reflects the co-expression of a ligand in one cell type and its receptor in another, calculated from mean expression values at the pseudobulk level. The chord diagram depicts the number of significant LR interactions between source and target cell types.

bioRxiv preprint doi: https://doi.org/10.64898/2026.04.28.721379; this version posted May 1, 2026. The copyright holder for this preprint (which was not certified by peer review) is the author/funder, who has granted bioRxiv a license to display the preprint in perpetuity. It is made available under aCC-BY 4.0 International license.

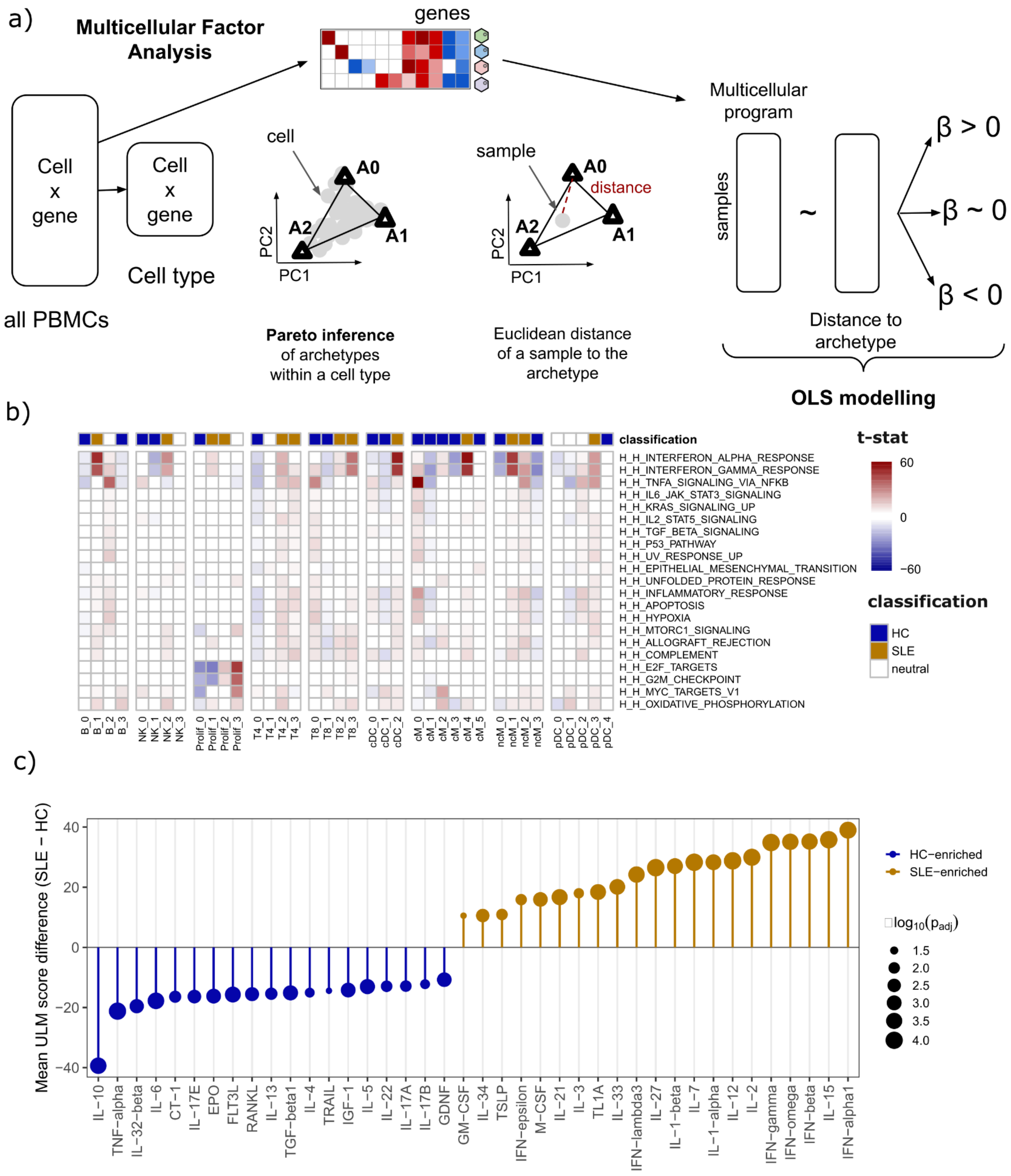

Supplementary Figure S5- Archetypal analysis

(A) Overview of the methodology to link multicellular programs to archetypal cell states across patients. Within each cell type, single-cell expression profiles are used to identify

bioRxiv preprint doi: https://doi.org/10.64898/2026.04.28.721379; this version posted May 1, 2026. The copyright holder for this preprint (which was not certified by peer review) is the author/funder, who has granted bioRxiv a license to display the preprint in perpetuity. It is made available under aCC-BY 4.0 International license.

archetypes using Pareto task inference, and each patient summary is projected onto the archetype simplex. Euclidean distances from each patient to each archetype are then computed. Finally, sample multicellular program scores (from IFN program) are modeled as a function of distance to each archetype using ordinary least squares. Negative regression coefficient indicates that higher program loading is associated with closer proximity to the corresponding archetype, whereas a positive coefficient indicates association with greater distance. (B) Hallmarks enrichment across all archetypes. Heatmap of ULM enrichment scores (t-values) for most enriched Hallmarks across all archetypes (columns), grouped by cell type. Only Hallmarks–archetype pairs with adjusted $P < 10^{-6}$ are shown (non-significant entries set to zero). Columns are ordered by cell type; vertical gaps separate cell types. Top annotation indicates archetype classification: SLE-associated (orange), Healthy-associated (blue), or neutral (gray), based on the regression of archetype proximity against IFN program scores (FDR < 0.05). Rows are hierarchically clustered. Color scale represents t-values: red, positive enrichment; blue, negative enrichment; white, non-significant or zero. (C) Differential cytokine enrichment between SLE- and HC-associated archetypes. For each cytokine, the mean ULM enrichment score was computed separately across SLE-associated and HC-associated archetypes, and the difference (SLE minus HC) is shown on the y-axis. Significance was assessed by Wilcoxon rank-sum test with Benjamini–Hochberg correction; top 40 most significant cytokines with adjusted $P < 0.05$ are displayed. Point size encodes $-\log_{10}$(adjusted P-value). Orange, SLE-enriched cytokines; blue, HC-enriched cytokines.

bioRxiv preprint doi: https://doi.org/10.64898/2026.04.28.721379; this version posted May 1, 2026. The copyright holder for this preprint (which was not certified by peer review) is the author/funder, who has granted bioRxiv a license to display the preprint in perpetuity. It is made available under aCC-BY 4.0 International license.

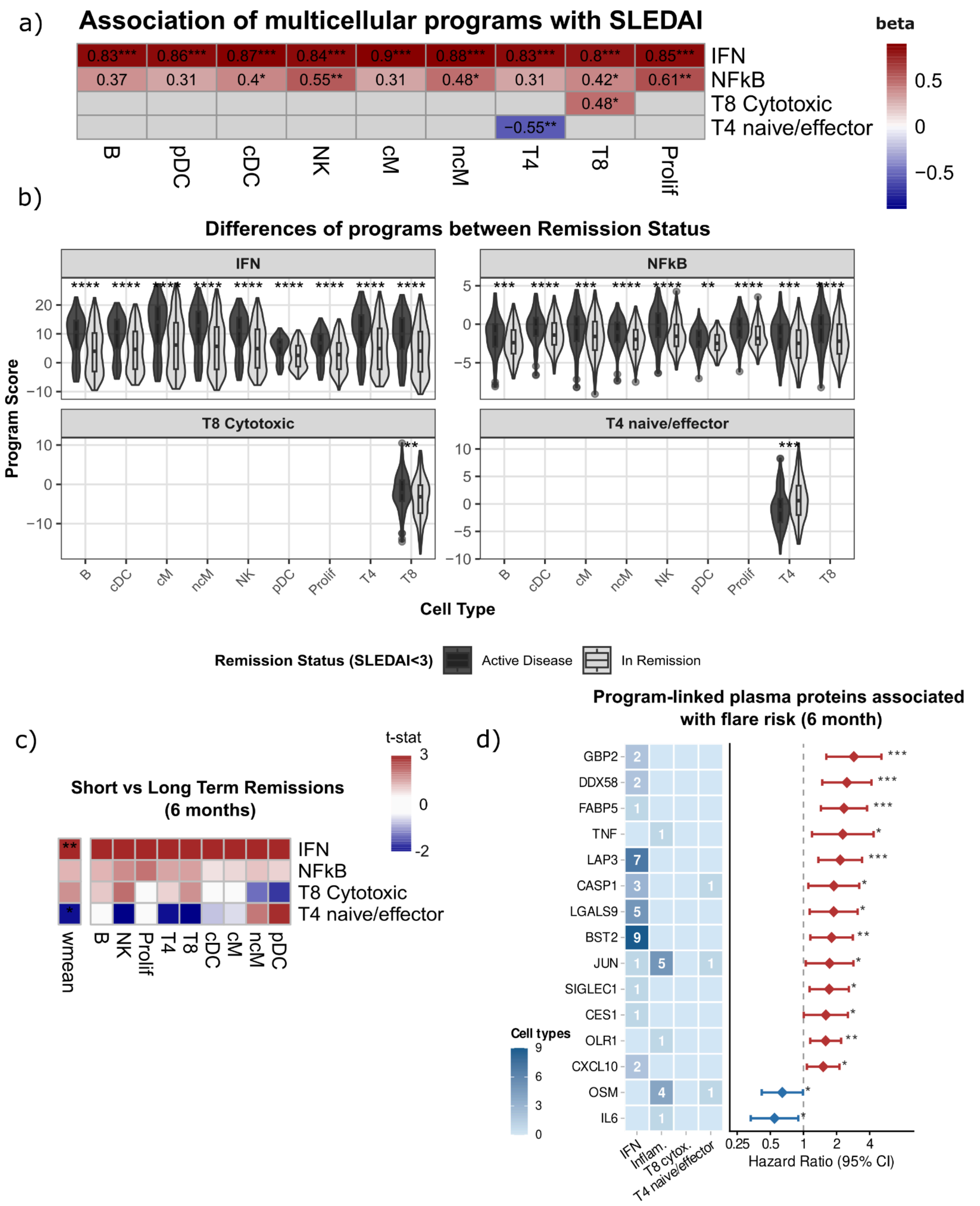

Supplementary Figure S6 - Flare prediction

(A) Heatmap showing the association between multicellular program scores and SLEDAI in the longitudinal adult SLE cohort. Each cell displays the standardized regression coefficient

bioRxiv preprint doi: https://doi.org/10.64898/2026.04.28.721379; this version posted May 1, 2026. The copyright holder for this preprint (which was not certified by peer review) is the author/funder, who has granted bioRxiv a license to display the preprint in perpetuity. It is made available under aCC-BY 4.0 International license.

(β) from a linear mixed-effects model. Rows correspond to the four retained multicellular programs and columns to immune cell types. Grey cells indicate program–cell-type combinations excluded from the model due to cell-type specificity of the program. Asterisks denote statistical significance after Benjamini–Hochberg correction: *p < 0.05, **p < 0.01, ***p < 0.001. (B) Violin plots showing the distribution of multicellular program scores across cell types, stratified by active (SLEDAI ≥ 3) vs. remission disease (SLEDAI < 3). Statistical comparisons between groups were performed using the Wilcoxon rank-sum test; significance levels are shown above each comparison (ns: not significant, *p < 0.05, **p < 0.01, ***p < 0.001). (C) Heatmaps showing differential immune program activity between remission visits that preceded a clinical flare (short-term remission) and those with sustained disease control (long-term remission), at a 6-month prediction time frame. For each program–cell type combination, differences were assessed by two-sample t-test; colour represents the t-statistic, with positive values indicating higher program activity in short-term (pre-flare) visits. The top row ("wmean") summarises a weighted mean across cell types, where weights are proportional to the variance explained (R^2) by each factor in each cell type as estimated by the multicellular factor model. P-values were corrected for multiple comparisons using the Holm procedure; asterisks denote significance: *p < 0.05, **p < 0.01, ***p < 0.001. (D) Combined plots showing genes significantly associated with flare risk at a 6-month prediction time frame, that overlap with top gene signatures of the multicellular programs and were detected in blood proteomic cohort [ref] upregulated in SLE patients. Left panel (heatmap): for each gene, the number of cell types in which it constitutes a top gene of the respective program (IFN, NFkB, T8 Cytotoxic, T4 naive/effector). Right panel (forest plot): hazard ratio (HR, 95% CI) from univariate Cox proportional hazards models, estimating the association between gene expression and time to flare. Proteins are ordered by HR. Red indicates proteins associated with increased flare risk (HR ≥ 1, p < 0.05); blue indicates protective associations (HR < 1, p < 0.05); grey indicates non-significant associations. Asterisks denote significance: *p < 0.05, **p < 0.01, ***p < 0.001.

bioRxiv preprint doi: https://doi.org/10.64898/2026.04.28.721379; this version posted May 1, 2026. The copyright holder for this preprint (which was not certified by peer review) is the author/funder, who has granted bioRxiv a license to display the preprint in perpetuity. It is made available under aCC-BY 4.0 International license.

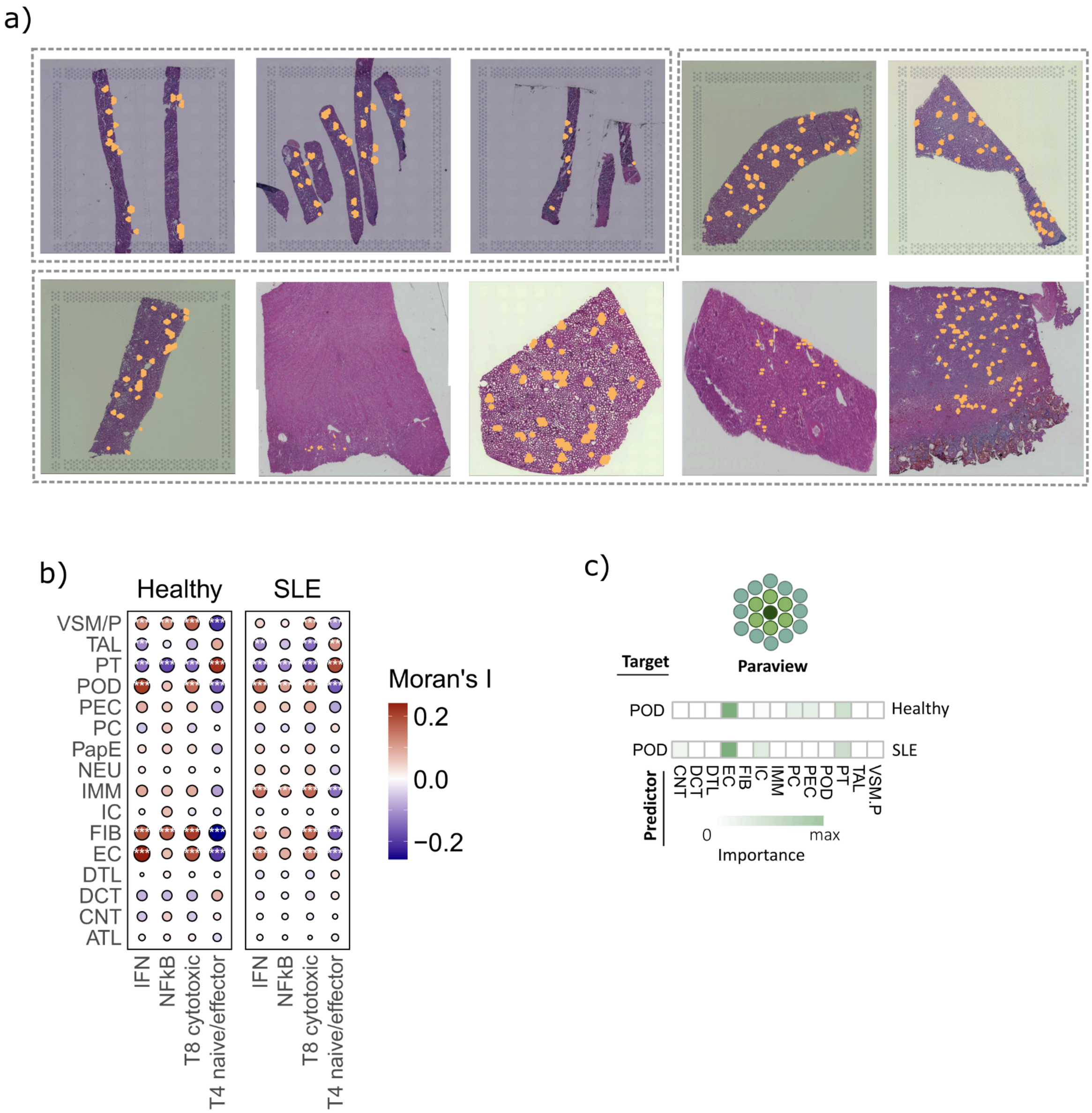

Supplementary Figure S7 - Spatial analysis of cell type composition in kidney samples.

(A) Visium 10x spatial transcriptomics slides from SLE (top row, first three) and healthy control kidneys (remaining slides) included in the analysis. Orange dots denote spatial spots annotated as glomeruli. (B) Spatial correlation between multicellular program activities and predicted cell-type abundances between conditions, quantified using bivariate Moran's I. Moran's I values were averaged across slides per condition (SLE, Healthy), and statistical significance was assessed by combining per-sample permutation-derived p-values using

bioRxiv preprint doi: https://doi.org/10.64898/2026.04.28.721379; this version posted May 1, 2026. The copyright holder for this preprint (which was not certified by peer review) is the author/funder, who has granted bioRxiv a license to display the preprint in perpetuity. It is made available under aCC-BY 4.0 International license.

Fisher's method, followed by Benjamini-Hochberg FDR correction. Dot size represents the magnitude of spatial association (|Moran's I|); color indicates directionality (red = positive, blue = negative). Asterisks denote associations that are simultaneously statistically significant (FDR < 0.05) and exceed a minimum effect size (|Moran's I| ≥ 0.10). (C) Variable importance from the paraview model for predicting key glomerular cell types (endothelial cells, EC; podocytes, POD). Higher importance values indicate stronger spatial influence from neighboring regions.
